# Switchable Broadband-to-Tunable Narrowband Magnetic Probe for Near-Field Measurements

**DOI:** 10.3390/s22197601

**Published:** 2022-10-07

**Authors:** Abdulghafor A. Abdulhameed, Zdeněk Kubík

**Affiliations:** 1 Department of Electronics and Information Technology, Faculty of Electrical Engineering, University of West Bohemia, 301 00 Pilsen, Czech Republic; 2 Department of Electrical Techniques, Qurna Technique Institute, Southern Technical University, Basra 61001, Iraq

**Keywords:** broadband, EMC, LC equivalent circuit, LGR magnetic probe, narrowband, PIN diode, varactor diode

## Abstract

This paper presents a printed magnetic probe that can switch from broadband to tunable narrowband for near-field measurement. In the early design stage, we created a printed loop gap resonator as a magnetic reference sensor for the pre-compliance test in a band up to 6 GHz. Consequently, the results showed a good response in terms of the S11 and S21 parameters of the proposed probe compared with the commercial magnetic sensor XF-R 3-1. The source noise might spread among different frequency bands, making the broadband magnetic probe the closest choice for estimating the magnetic field in the near-field region. Unfortunately, broadband magnetic probes have lower sensitivity than narrowband ones. One of the solutions to get high sensitivity is to connect the LNA to the output of the passive magnetic sensor. This work proposes a novel method to solve this issue using a PIN diode to change the broadband status into a high sensitivity narrowband status and then tune this narrowband across the most critical applications such as 3.5 GHz, 3.75 GHz, 4.8 GHz, and 5.2 GHz with the help of a varactor diode. Compared to the broadband status, an improvement of more than 10 dB has been obtained across all these wireless bands. Furthermore, the proposed structure’s isolation between the electrical and magnetic fields is about 13 dB.

## 1. Introduction

The massive growth in radio communications has led to occupation of the spectrum by radio-electronic techniques that spread over a wide bandwidth. Therefore, ultrawideband (UWB) technology was the best choice since it has a band wide enough to cover essential applications like Worldwide interoperability for microwave access (WiMAX), Wireless local area network (WLAN), Personal Area Network (PAN), and satellite communications [1]. The UWB technology has two problems; the interference between the UWB and the other single bands operating at the same frequency, and the cost of providing the UWB spectrum for using just one or two narrow bands simultaneously. The cognitive radio system solved these problems with the help of two antennas—a UWB antenna for sensing idle bands and a reconfigurable antenna for communication in these bands [2,3,4,5,6]. 

**Figure 1:**
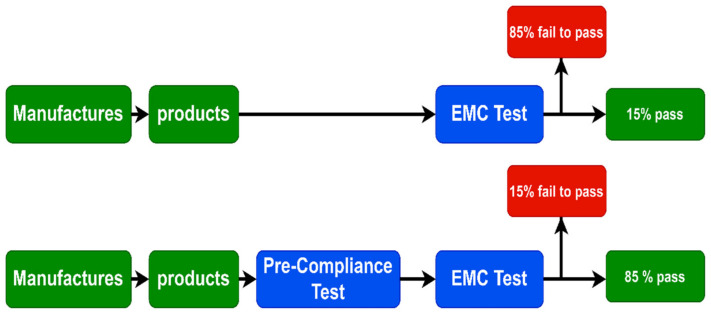
Block diagram presenting the EMC test with and without the pre-compliance test.

Electronic circuits are considered a potential source of noise to well-known applications like Global System for Mobile (GSM), Wireless fidelity (Wi-Fi), WiMAX, PAN, and WLAN due to them having high-speed switching circuits embedded in their printed board (PCB). Therefore, new pre-compliance products are subject to electromagnetic compatibility (EMC) tests to ensure they are compatible with the other parts in the same unit and do not affect the other devices before releasing them to the markets. Using the pre-compliance test as an intermediate stage with the EMC test increases the probability of successfully passing the test to 85% instead of 15%, as shown in Figure 1 [7].

The United Kingdom (UK) government adopted laws in 1992 to force the manufacturers of all electronic devices or appliances to submit their products to the EMC test before releasing them to the markets. One of the mandatory parts of the EMC test is the electromagnetic sensors (reference antenna and electric/magnetic probe) [8,9,10]. The EMC measurement consists of two methods; the far-field method uses the reference antenna to estimate/radiate the electrical field strength (E_max_) and it is recommended when the level of source interference is significantly high [11]. In contrast, when the source interference is relatively small, the best choice is to utilize magnetic or electric probes in the proximity area above the Equipment Under Test (EUT) [12]. The electric probes suffer from the disturbance of the EUT radiation emitted by the sensor itself when its response approaches the resonance frequency and becomes frequency-dependent [13].

The classical magnetic probe can be easily formed by hand into different shapes like circular and square loops, but it suffers from low accuracy due to its relatively large size [14]. On the other hand, printed magnetic probes have been grown using printed circuit board technology (PCB) in the last two decades, with many advantages like compact size, high accuracy, multilayers, and fabricating ease [15]. The four critical parameters to design any magnetic probe are bandwidth, spatial resolution, sensitivity, and isolation [16]. Several printed magnetic probe structures have been proposed in the last two decades to fulfill these design parameters.

The sensitivity measures the strength of the induced magnetic field of the designed probe above the reference plane. Typically, there is a 2 mm reference height between the designed probe and the reference board when measuring the sensitivity. This factor can be estimated using two methods; first, it represents the mutual coupling between the designed magnetic probe and the reference board (S21 parameter). Second, it can be estimated by measuring the two minimum points of the magnetic field distribution, which indicate the detection accuracy.

The broadband magnetic probe covers a wide bandwidth, making it an excellent choice for estimating the magnetic field surrounding electronic circuits. Unfortunately, it suffers from low sensitivity compared to narrowband magnetic probes [17,18]. Connecting a low noise amplifier (LNA) at the output of the passive magnetic probe to enhance the sensitivity is one of the solutions to overcome the broadband probe issue [19]. A novel rectangular broadband (up to 7 GHz) magnetic probe was presented in Ref. [20]. The electromagnetic emission from the ground plane was effectively suppressed by adopting a tapered transition between the rectangular loop and the coplanar waveguide 50 Ω transmission line. Consequently, this transition eliminated the capacitive effect and enhanced the operation bandwidth compared to the conventional probe with the same covering band. 

A printed novel circular broadband (up to 9 GHz) magnetic probe was proposed to serve as a sensor for near-field measurement [21]. This probe was based on the fiberglass microwave substrate FR-4 with relative permittivity $\varepsilon_{r}\;$= 4.4 and thickness h = 0.8 mm. A group of quasi-periodic notches was engraved on the feeding line to work as a band-stop filter to eliminate the probe’s self-resonance. Moreover, the proposed structure accurately detected the magnetic field with high isolation by suppressing the unwanted electrical field.

A magnetic probe with four ultra-band layers up to 20 GHz was proposed in [22]. The via fence technique was employed to suppress the resonance at the operating frequency, making the S21 parameter curve more smooth. Despite utilizing the via fence technique, the resulting probe does not have a flat transition for the whole frequency band. For instance, the sensitivity in terms of S21 parameters was about −40 dB at 2 GHz, then reached −30 dB at 12 GHz, and went back down again to −40 dB at 20 GHz. Ref. [23] presented an ultra-wideband miniature magnetic probe with a high spatial resolution. The operating frequency was extended up to 30 GHz with a high sensitivity of −30 dB by reducing the parasitic inductance and parasitic capacitance using small loops and tapered transition techniques. The high spatial resolution of this probe achieved with a 250 µm × 250 µm aperture slot enabled it to detect the magnetic field in small devices. However, this probe suffered from high fluctuations in sensitivity across the whole operating band up to 30 GHz. A small broadband shielded magnetic sensor for near-field measurement was presented in [24]. The structure was based on an FR-4 substrate and consisted of four stick-up layers. Furthermore, the relative sensitivity was enhanced by about 5 dB for the covering band from 100 MHz to 6 GHz using an inverted G-shape inductive line. Via fence and metal canned techniques were utilized simultaneously to suppress unwanted electrical fields and achieve high isolation. 

Narrowband probes usually have high sensitivity at the specific working frequency. A highly sensitive resonance magnetic probe for measuring radio-frequency interference was demonstrated in [25]. Incorporating the LC resonance circuit in differential mode with Marchand balun exhibited a sensitivity improvement of about 8.63 dB at 1.57 GHz. Moreover, this probe showed its validity when it was used to detect the radiation emitted from PCB traces and cell phones in the real world. The authors of Ref. [26] proposed design procedures for an electric probe working at 1.57 GHz. This structure consisted of LC resonators followed by quarter-wavelength transformers to achieve optimal power transformation at the resonance frequency. This probe was dedicated to determining the changing voltage source of the device under test. The sensitivity enhancement was 6.6 dB compared with the result of the broadband equivalent size probe. Ref. [27] presented modeling and investigation of a high-sensitivity narrowband magnetic probe’s design. The use of an LC resonator afforded a sensitivity of about 20 dB compared to the conventional equivalent magnetic sensor.

The source noise might spread among multi-frequency bands over the device under test (DUT). The electromagnetic interference at multiple frequency bands can be estimated using low-sensitivity broadband probes or multi-narrowband high-sensitivity magnetic probes. A tunable resonance magnetic probe was designed, fabricated, and investigated in [28]. This probe used a varactor diode to switch the frequency bands among the GSM900, UMTS, and GPS applications to improve the sensitivity by 7–9 dB compared to the sensitivity results of a broadband magnetic probe of equivalent size. The authors used Advance Design System (ADS) software to model the equivalent circuit of this probe. Furthermore, the measurement results showed good agreement with the simulation results from the ADS software.

This work presents further advantages than what has been reported in the previous literature reviewed by designing, modeling, and fabricating a switchable broadband-to-multi-narrowband magnetic probe. Switching is achieved using a PIN diode for broadband-to-single-band transition. Simultaneously, the varactor diode is utilized to tune the obtained single band to the most influential bands like WiMAX (3.5 GHz), mid-bandwidth (3.6–3.8) GHz for 5G applications, PAN (4.8 GHz), and WLAN (5.2 GHz). This article is organized as follows. Section 2: Describes the design, modeling, and analysis of a switchable magnetic probe. The basing circuit for this probe is illustrated in Section 3. Section 4: Briefly discusses the design and modeling of the equivalent circuit in AWR Software. Section 5: Demonstrates the isolation and sensitivity results compared with the literature reviewed. Finally, Section 6: Presents a brief conclusion.

## 2. Magnetic Probe Design

**Figure 2:**
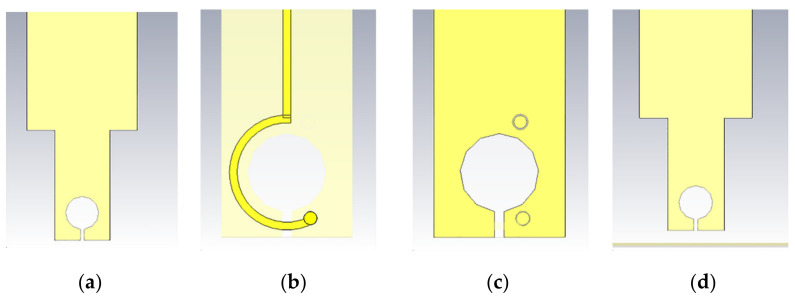
Magnetic probe layers: (**a**) Top layer (ground); (**b**) detailed view of the stripline layer; (**c**) detailed view of the symmetry-maintaining layer; (**d**) bottom layer (ground).

**Figure 3:**
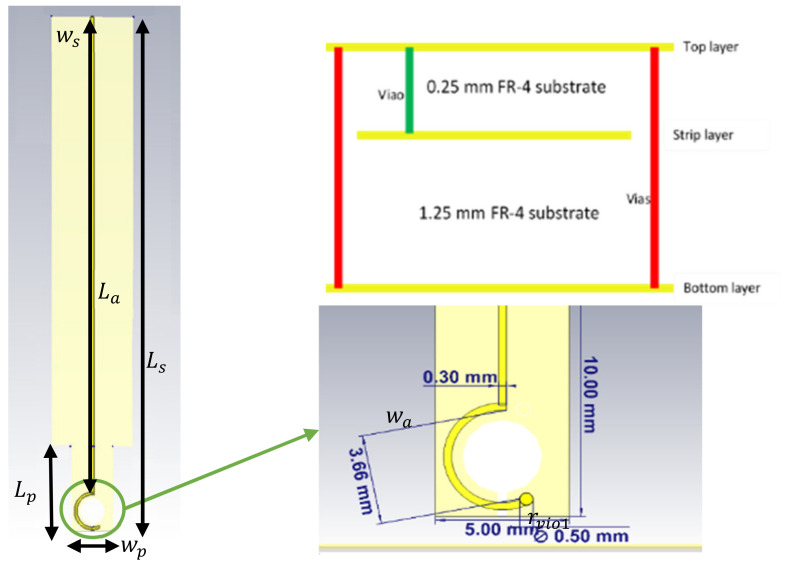
Front view of the magnetic probe with all dimensions.

The proposed magnetic probe consists of four stick-up layers; the first and fourth layers are employed as shielded ground layers for isolation purposes, the second layer stands for the strip loop and transmission line, and the third layer is dedicated to maintaining the symmetry of the structure. This probe is based on an FR-4 substrate with a relative permittivity of $\varepsilon_{r}=4.3$ and a loss tangent tanδ=0.025. Figure 2 shows the geometrical shape of the probe’s layers, while the dimensions are illustrated in Figure 3.

**Figure 4:**
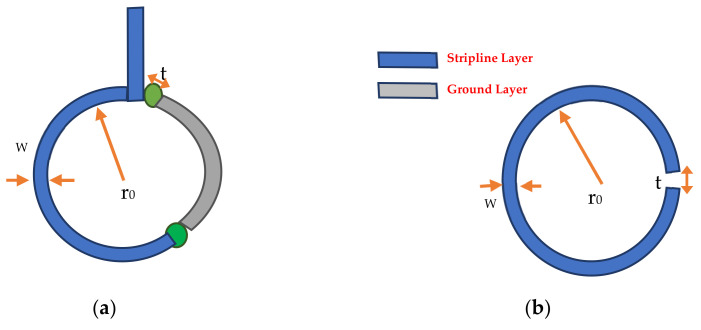
Magnetic probe layers: (**a**) proposed probe; (**b**) loop gap resonator.

The structure of the proposed probe in the typical case (broadband response) can be represented by a Loop Gap Resonator (LGR), as shown in Figure 4. The resonance frequency of the LGR is based on the capacitor of the gap and the loop’s inductor, which can be calculated mathematically using Equation (1) [29].

(1) $$C=\varepsilon_{0}\frac{w\;l}{n\;t},$$

(2) $$L=\mu_{0}\frac{\pi r^{2}}{l}.$$

Since,

(3) $$f_{0}=\frac{1}{2\pi \sqrt{LC}}.$$

Substituting (1) and (2) in (3) yields:

(4) $$f_{0}=\frac{v_{0}}{2\pi r}\sqrt{\frac{n\;t}{\pi \;w}},$$

where *n* is the number of gaps in the loop, *w* is the strip width in mm, *r* represents the probe’s radius in mm, and *t* is the gap width in mm and equal to (2 × $r_{via2}$).

**Figure 5:**
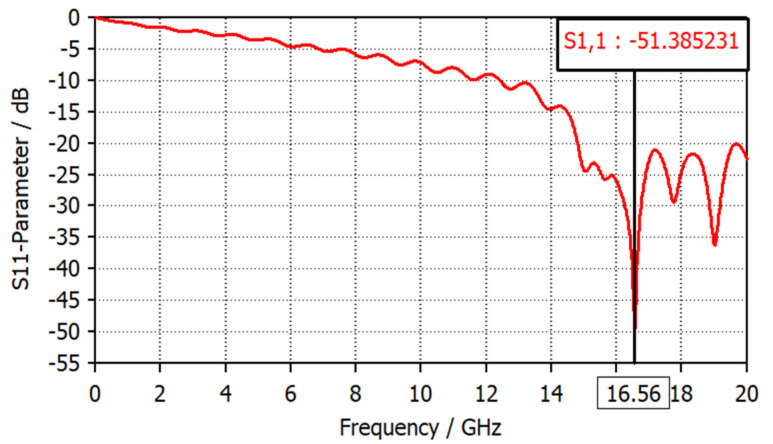
Reflection coefficient versus frequency of the LGR.

The theoretical resonance frequency of the LGR is computed by substituting the probe’s parameter values in Equation (4), where w=0.56 mm, r=1.7 mm and *t* = 0.5 mm. Consequently, the theoretical result of the resonance frequency coincided with the simulated S11 parameter obtained from the CST EM Microwave Studio [30], where $f_{0}=\frac{3\times 10^{8}}{2\pi \times 1.7\times 10^{-3}}\sqrt{\frac{0.5\times 10^{-3}}{\pi \times 0.56\times 10^{-3}}}$ = 14.9 GHz. The simulated reflection coefficient is shown in Figure 5, while the overall dimensions of the proposed probe are listed in Table 1.

**Figure 6:**
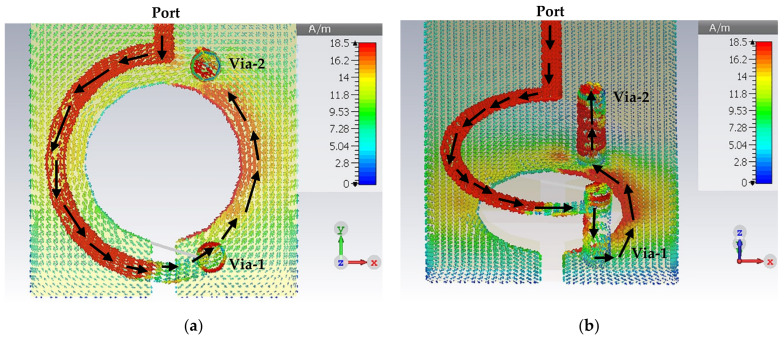
The surface current distribution of the broadband magnetic probe: (**a**) top view; (**b**) perspective view.

Figure 6 presents the surface current distribution of the designed LGR probe in 2D and 3D views, which is an essential criterion to gain deep insight into the broadband probe’s resonance behavior. It can be seen that the power flows from the port toward the loop through the transmission line, then moves to the other side through via-1 and back to complete the open circle, as evidenced by the black arrows.

The S11 parameter describes the electrical characteristic of the antenna, and determines its impedance bandwidth. Furthermore, both the S11 parameter and S21 parameter are critical factors in evaluating the magnetic probe characteristics; the S21 parameter measures the strengths of the magnetic field and the electrical field in the vertical and horizontal directions, respectively. At the same time, the S11 parameter describes the resonance characteristics of the magnetic probe. Unlike the antenna, the magnetic probe operation bandwidth is computed by taking the −3 dB value of the reflection coefficient curve [31].

**Figure 7:**
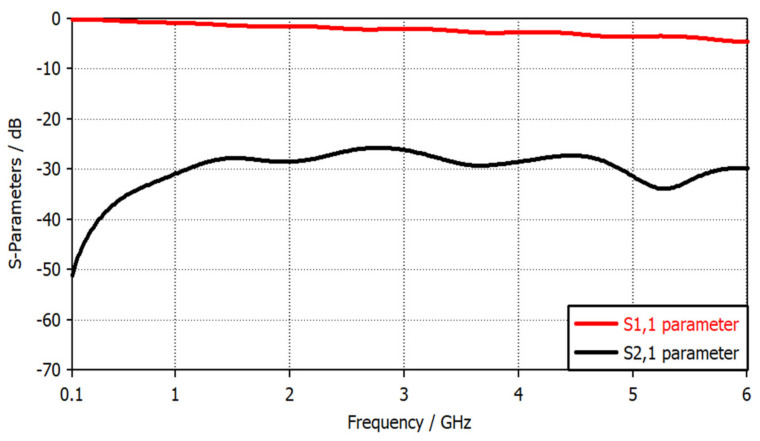
S-parameters of the broadband magnetic probe versus frequency.

**Figure 8:**
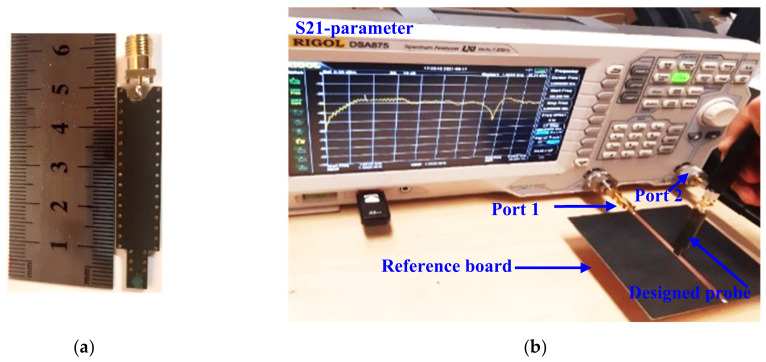
Prototype of the magnetic probe: (**a**) dimensions of the probe; (**b**) S21-parameter measurement setup.

**Figure 9:**
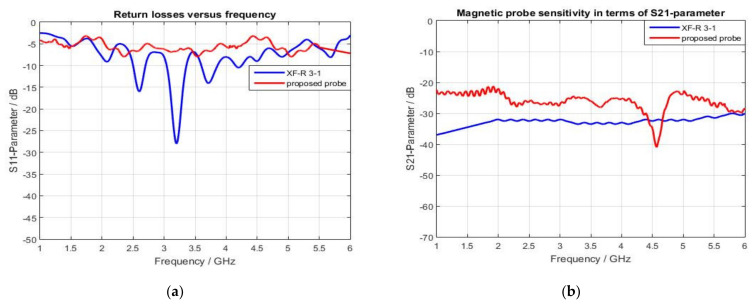
S-parameters of the proposed magnetic probe compared with the commercial XF-R 3-1: (**a**) S11 parameter; (**b**) S21 parameter.

Figure 7 shows the simulated S-parameters of the proposed magnetic probe with a 2 mm height above the trace of the microstrip reference board. It can be seen that this probe has high sensitivity (as S21 > −27 dB) in the 1 GHz to 3.4 GHz band, and starts losing its sensitivity (as S11 < −3 dB) beginning at 3.5 GHz and beyond. The proposed broadband magnetic probe has been fabricated and tested with the help of a Network Analyzer, a Directional Coupler, and a reference board, as shown in Figure 8b. Moreover, the achieved results have been validated by comparing them to the results obtained with the commercial broadband magnetic probe XF-R 3-1 that is offered for sale [32], as shown in Figure 9.

The proposed probe performs better than the XF-R 3-1 magnetic probe in terms of sensitivity using S11 and S21 parameters. For instance, the sensitivity of the proposed magnetic sensor is better than the commercial probe, which makes sense because the spatial resolution of the proposed magnetic sensor is 1.6 mm compared to 1 mm for the XF-R 3-1 probe.

The 3.5–6 GHz band covers many radio frequency applications like WiMAX at 3.5 GHz, mid-bandwidth 3.6–3.8 GHz for 5G applications, PAN at 4.8 GHz, and WLAN at 5.2 GHz. Therefore, one of the benefits of increasing the broadband magnetic probe’s sensitivity is accurately estimating the magnetic field interference with these applications. The switchable magnetic probe can solve this issue using the different switching techniques presented in [33,34,35].

**Figure 10:**
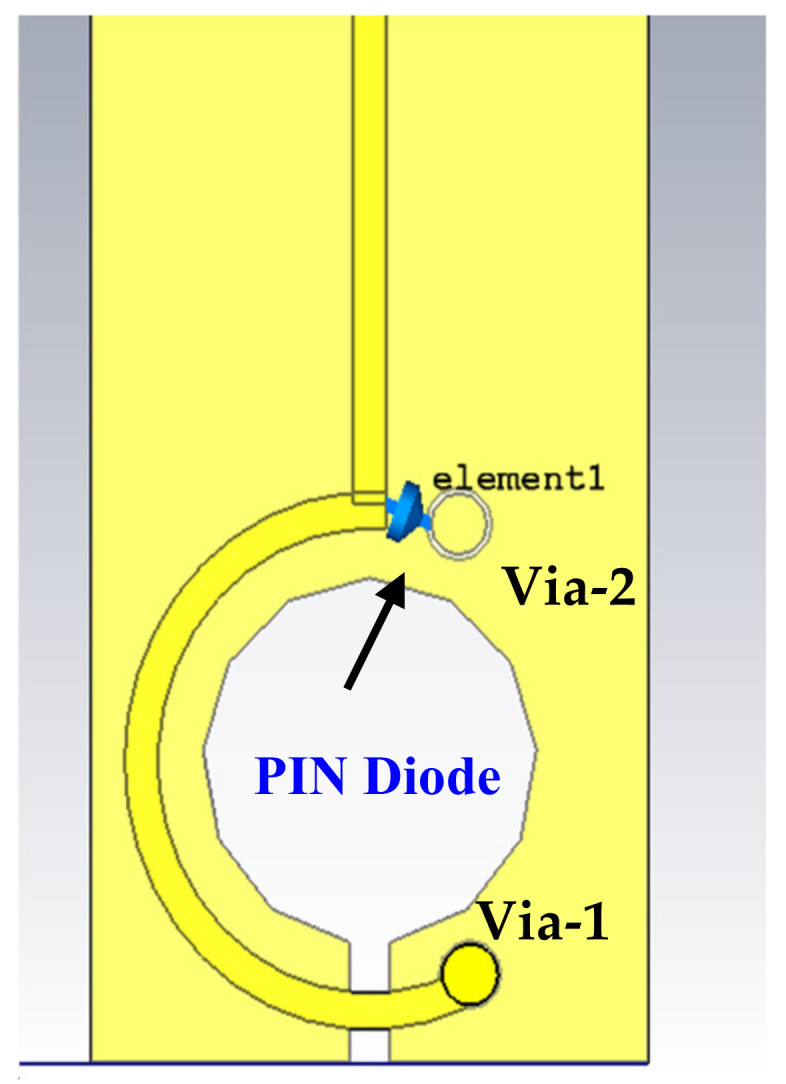
Introducing the PIN diode in the broadband magnetic probe.

A PIN diode type SMP1320-079LF has been utilized to switch the resonance behavior from broadband to high-sensitivity narrowband. Simultaneously, a varactor diode is employed to tune the achieved narrowband resonance frequency by changing the parasitic capacitance value. The PIN diode is inserted between the beginning point of the loop and the via-2. Thus, it connects the other side of the circular arc with the ground layers, as shown in Figure 10.

**Figure 11:**
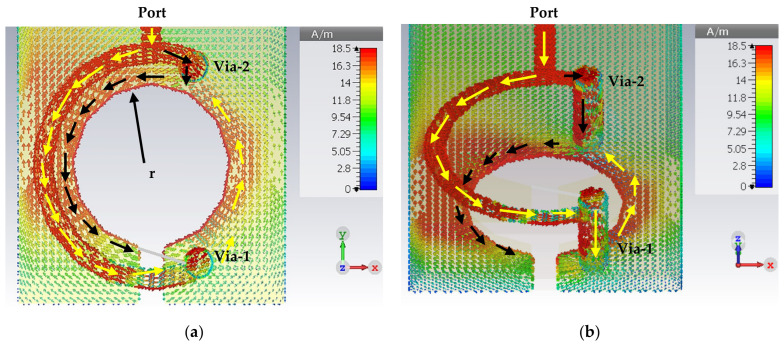
The surface current distribution of the narrowband magnetic probe: (**a**) top view; (**b**) perspective view.

Using the PIN diode will create another surface current direction; the first was from the port toward the loop and through the via-1 toward the via-2, as shown by the yellow arrows in Figure 11. At the same time, the second direction will start from the microstrip line through the via-2 toward the via-1 on the other side of the loop, as is denoted by black arrows, hence forming a circle of surface currents, as shown in Figure 11. As the vias connect the top and bottom layers, there will be two slotted patch gap resonators, one at the top and the other at the bottom. After adding a PIN diode, the LGR was changed to two patch-slotted gap resonators (one on the top and the other on the bottom layers). Figure 11b depicts only the bottom layer to provide a clear view.

**Figure 12:**
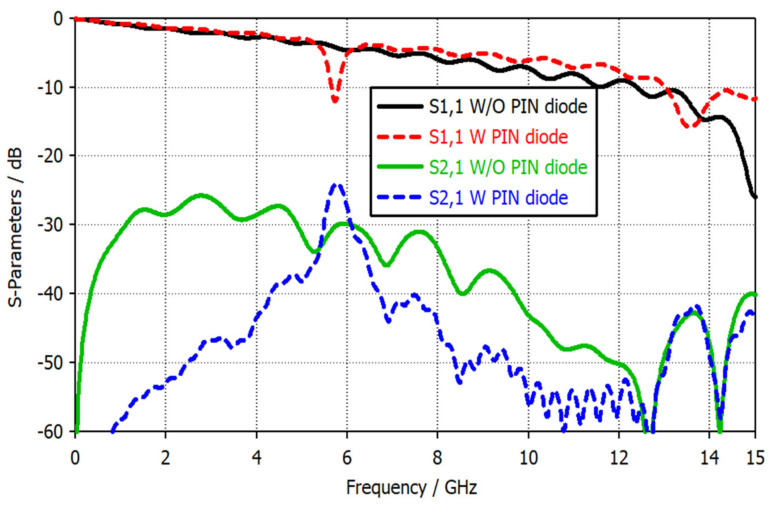
S-parameters of the proposed magnetic probe with and without using a PIN diode.

Moreover, the resonance frequency for this new structure can be calculated using the previous Equations (1–4). The calculated resonance frequency is 13 GHz, and since there are two resonators of the slotted patch gap, the probe shows another frequency band at 5.8 GHz, as shown by the red dashed curve in Figure 12. This narrowband magnetic probe has a high sensitivity enhancement (S21 parameter) of about 6 dB compared to a broadband magnetic probe.

**Figure 13:**
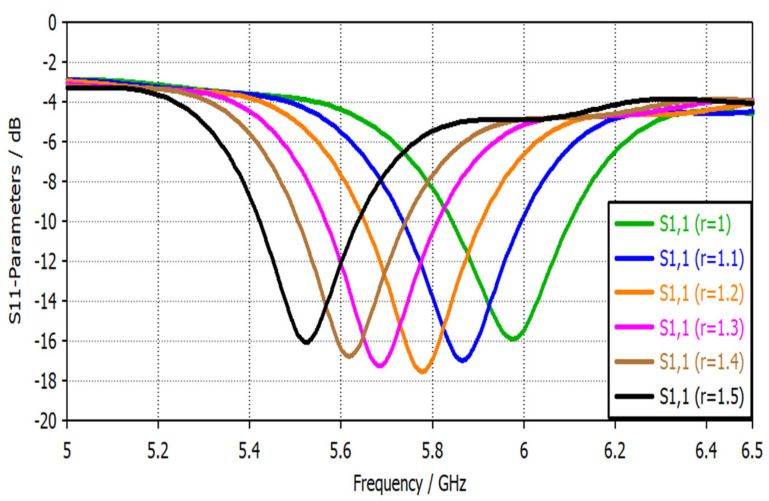
S11 parameters of the narrowband magnetic probe for different values of a slotted circle radius *r*.

**Figure 14:**
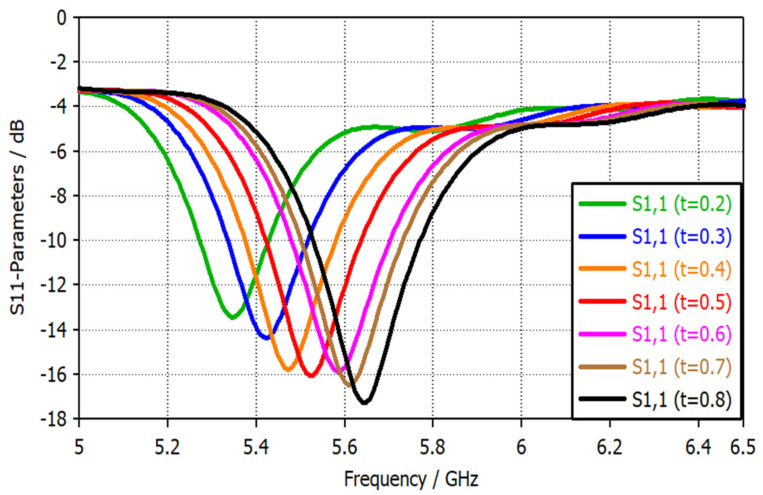
S11 parameters of the narrowband magnetic probe for different values of slotted gap t in mm.

The slotted circular gap radius *r* controls the resonance frequency location. For instance, increasing the radius *r* will shift the resonance frequency toward the lower bands, as shown in Figure 13. Moreover, the probe’s slotted gap width *t* significantly affected the resonance frequency. Figure 14 shows the reflection coefficient versus frequency for different values of the slotted gap width. Increasing the gap width *t* will increase the frequency since both the gap width *t* and the frequency *f* are directly proportional to each other, according to Equation (4).

The frequency shift achieved with different values of the gap width *t* has inspired the authors and led them to the next step by changing the position of the high sensitivity narrowband across several frequency bands like WiMAX, PAN, WLAN, etc.

**Figure 15:**
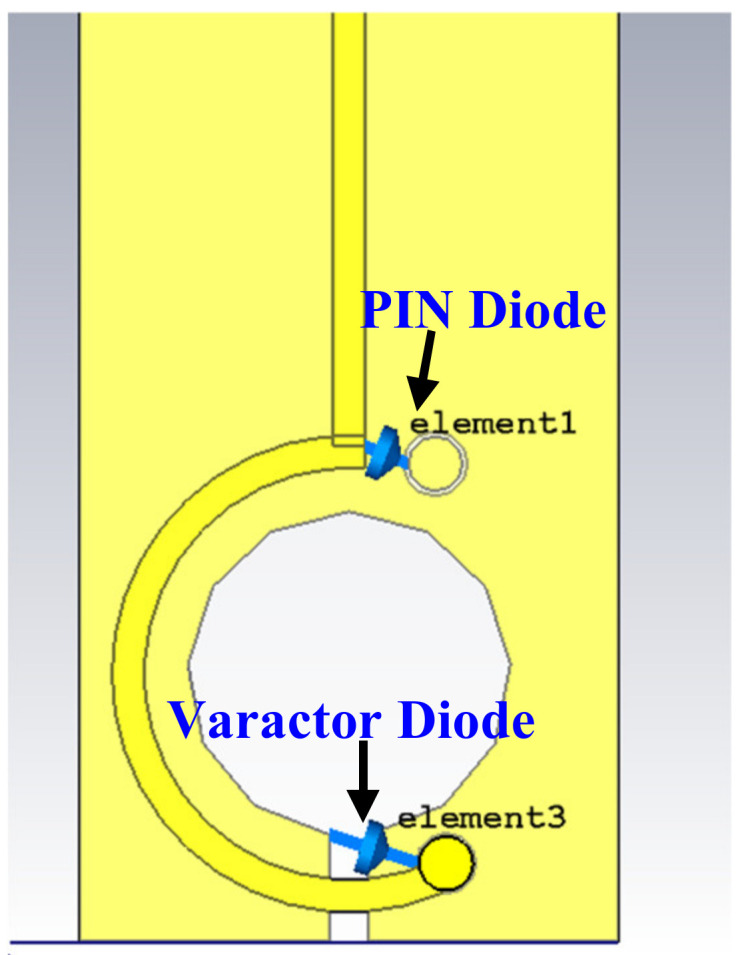
Use of the PIN diode and varactor diode in the magnetic probe.

**Figure 16:**
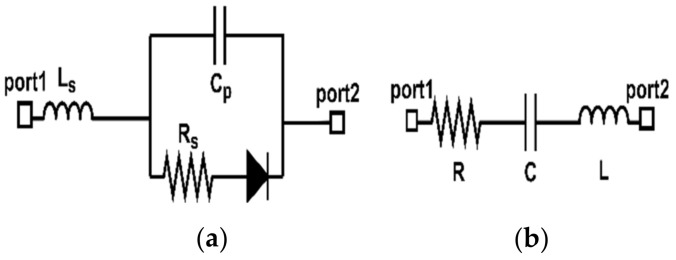
Varactor diode model: (**a**) spice model; (**b**) equivalent circuit.

The second step is to use a varactor diode (reverse bias voltage) between the via-1 and the far side of the slotted patch gap (across the opening gap). The varactor diode will change the parasitic capacitance value, hence changing the slot’s dimensions. Figure 15 depicts the geometrical shape upon inserting the varactor diode into the structure. Figure 16a shows the spice model of the varactor diode type SMV2201—040LF, *Ls* = 0.45 nH, *Cp* = 0.075 pF, and *Rs* = 5.41 Ω, while its equivalent circuit is shown in Figure 16b.

**Figure 17:**
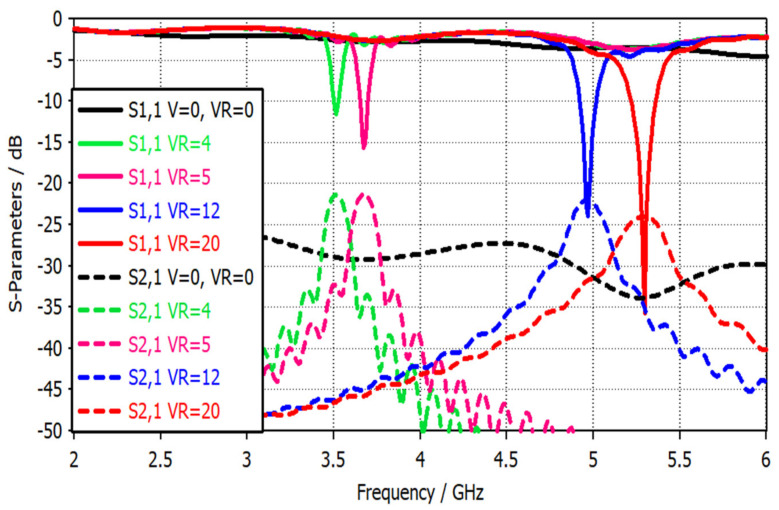
S-parameters with different PIN diode and varactor diode switching modes.

Figure 17 presents the simulated S-parameter results for both the broadband mode and tuned narrowband mode. The sensitivity enhancement of the tuned narrowband mode is about 10 dB compared to the broadband mode through all frequency bands.

**Figure 18:**
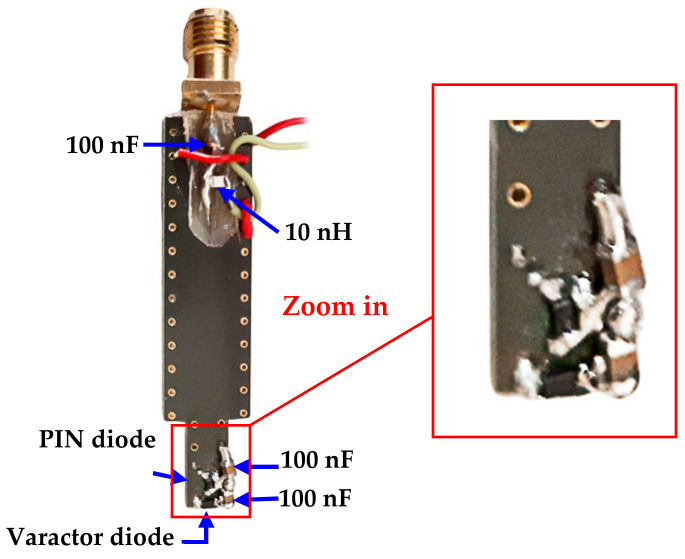
The fabricated shape of the magnetic probe from the Pragoboard company.

Based on the SMV2201-040LF datasheet from the Skyworks website, the parasitic capacitance value will decrease as the reverse biasing voltage increases. This probe has been fabricated by the Pragoboard company [36]. Figure 18 demonstrates the manufactured shape fabricated by the Pragoboard company.

## 3. Biasing Circuit

**Figure 19:**
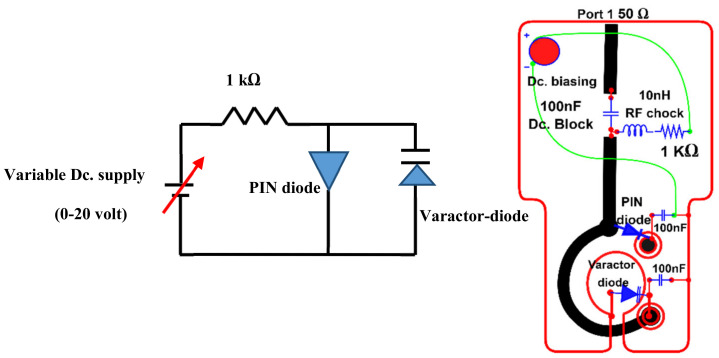
The biasing circuit for PIN and varactor diodes.

This work has two active elements that need biasing; the PIN diode and the varactor diode. The selected PIN diode SMP1320-079LF has a slight forward resistance *Rf =* 0.9 Ω at 10 mA and a reverse biasing capacitance *Cr =* 0.3 pF. On the other hand, the varactor diode type SMV2201-040LF we utilized has a capacitance range *C* = (0.23–2.22 pF). Figure 19 presents the biasing circuit for both the PIN and varactor diodes. Both DC current and RF currents will share the same feeding line. Therefore, three essential passive elements are involved to ensure the compatibility of these two sources. The Lc=10 nH chocking inductor of prevents radiation leakage from passing through the biasing circuit since it works as a low-pass filter. A 100 nF blocking capacitor C1 is necessary to protect the network analyzer from the biasing current. Finally, other 100 nF blocking capacitors C2 and C3 are mandatory to prevent the diodes’ reverse biasing and protect them from the short circuit since they work as a high pass filter. 

The path connecting the via-1 and the via-2 is part of the ground plane layers and carries a DC current. However, this part is involved in the magnetic resonance process (part of patch-slotted gap resonators), which iswhy it is connected to the vias. Therefore, two 100 pF capacitors are employed to prevent the back DC current for each diode from passing through this radiated path and to protect the diode from the short circuit by the DC current. 

The PIN diode has two states, ON and OFF, to switch between the narrowband and broadband states. At the same time, the varactor diode has a tunable mode to shift the resonance frequency when the PIN diode is in the ON status. Table 2 lists the diode modes with a variable DC voltage supply.

**Figure 20:**
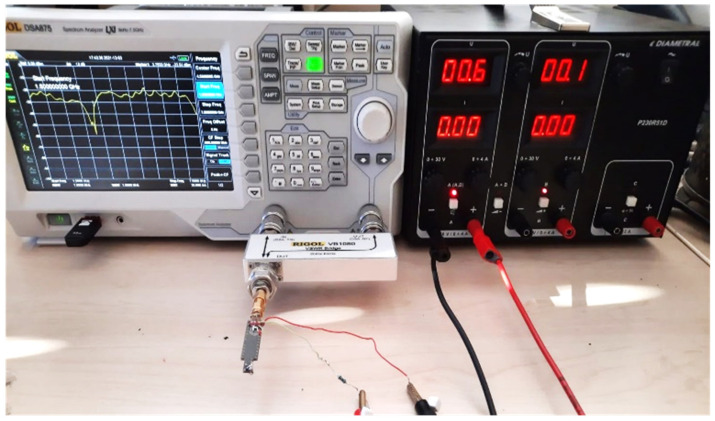
S11 parameter measurement with different biasing voltages.

One of the main advantages of near-field measurements is that they can perform in an ordinary laboratory with the necessary tools, unlike the far-field tests that affect the environment, which is why the latter needs chambers. The RIGOL DSA875 Spectrum Analyzer with the RIGOL VB 1032 directional coupler is utilized to measure the S11 parameter with the help of a DC voltage supply for biasing, as shown in Figure 20. Moreover, the sensitivity is achieved using the Spectrum Analyzer with the help of the printed reference board presented in [31].

**Figure 21:**
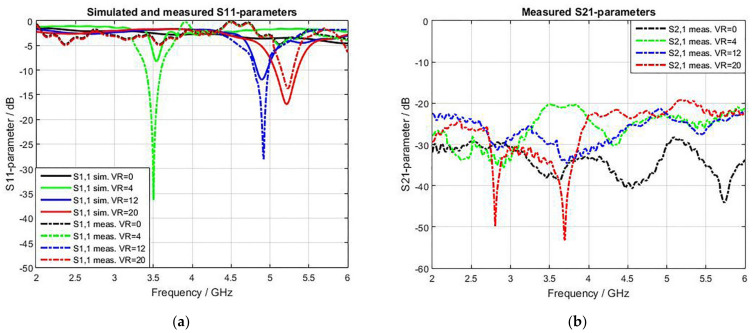
Simulated and measured S-parameters with different biasing voltage results: (**a**) S11 parameters; (**b**) S21 parameters.

Figure 21 presents the measured S-parameters for different switching modes. The resonance frequency bands achieved are three bands instead of four (missing a band at 3.75 GHz), which is due to the biasing voltage value (5 V) of band 3.75 GHz being close to that of band 3.5 GHz (4 V). However, the achieved measured band of 3.5 GHz has wider bandwidth than the simulation one due to the merging of these two adjacent bands, as demonstrated in Figure 21a.

Moreover, the sensitivity of the broadband state at VR = 0 V is lower than the sensitivity of the broadband probe in Figure 9b. The reason is that adding the PIN and varactor diodes will add some losses.

## 4. Equivalent Circuit of Magnetic Probe

The equivalent circuit of the proposed magnetic probe has been modeled and simulated using AWR Software [37]. The authors start by modeling the microstrip reference board with an input port of 50 Ω connected to parallel inductors. On the other hand, the parasitic elements (series inductor, radiation resistance, and parallel capacitor) represent the shielded loop. 

In this work, the value of the first inductor is chosen to be $L_{1}=$ 0.5 nH and this inductor is responsible for controlling the mutual coupling level at a specific frequency. In contrast, the values of the second inductor (self-inductor) and the self-capacitor are calculated using Equations (5) and (6), respectively. It is worth mentioning that the value of self-capacitance will be affected by the varactor diode’s weight of capacitance *C* = (0.23–2.22) pF in controlling the resonance frequency.

(5) $$L=\mu_{r}\mu_{o}r\left(\ln\left(\frac{8\times r}{w}\right)-2\right),$$

(6) $$C=\varepsilon_{r}\varepsilon_{0}r\;\left(ln\left(\frac{8\times r}{w}\right)-2\right),$$

where $\mu_{r}\;\mathrm{and}\;\mu_{o}$ are the relative and absolute permeabilities, $\varepsilon_{r}\;\mathrm{and}\;\varepsilon_{0}$ are the relative and absolute permittivities, and r and w are the radius and width of the loop with values of $r=1.7\times 10^{-3}\;\mathrm{mm}\;\mathrm{and}\;w=0.56\times 10^{-3}\;\mathrm{mm}$.

**Figure 22:**
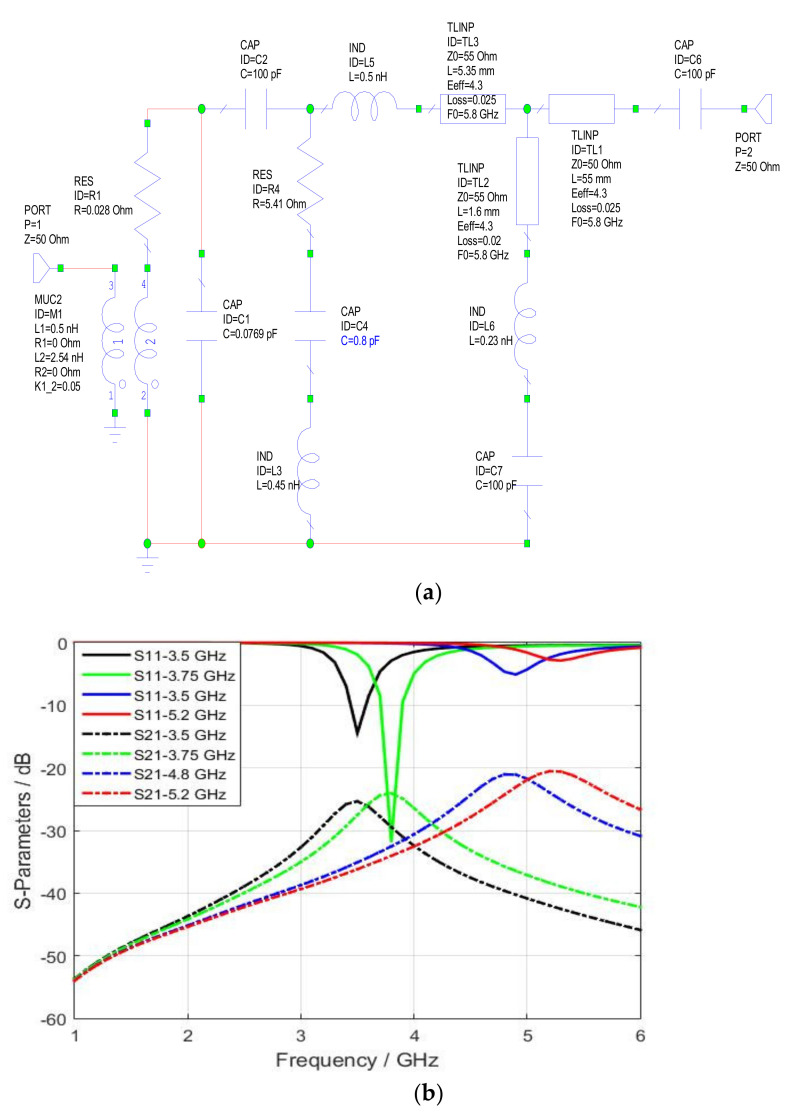
Modeling of the magnetic probe in AWR: (**a**) Equivalent circuit of the probe; (**b**) S-parameters with different switching modes.

The self-inductance and self-capacitance of the loop are equal to *L* = 2.45 nH and *C* = 0.07 pF by substituting the loop dimensions into Equations (5) and (6). A C = 100 pF blocking capacitor is inserted to isolate the high-frequency currents from the direct current of the varactor diode, which has one end connected to the blocking capacitor. In contrast, the second terminal is connected to the ground. An inductor with L = 0.5 nH and *R* = 55 Ω represents the part of the via- 1 connecting the varactor diode to the stripline and the 5.35 mm transmission line. Similarly, an inductor with *L* = 0.23 nH and *R* = 55 Ω represents the via-2 and the small piece of the transmission line connecting the PIN diode to the via-2. Another *C* = 100 pF blocking capacitor is utilized here to protect the probe from the DC current of the diode. The other path connects the arc to the input port through a 50 Ω transmission line. The equivalent circuit diagram obtained using AWR Software is shown in Figure 22a, while Figure 22b presents the frequency response of this equivalent circuit with different biasing values.

## 5. Isolation and Sensitivity

One of the critical steps in magnetic probe design is to measure the ability of the probe to suppress an undesirable electrical field. The transmission coefficient S21 parameter is utilized to represent the electrical field or the magnetic field, based on the direction of the sensor related to the reference microstrip trace. Thus, the magnetic field can be estimated when the sensor and the reference trace are in a parallel orientation. On the other hand, the orthogonality case offers the electrical field.

**Figure 23:**
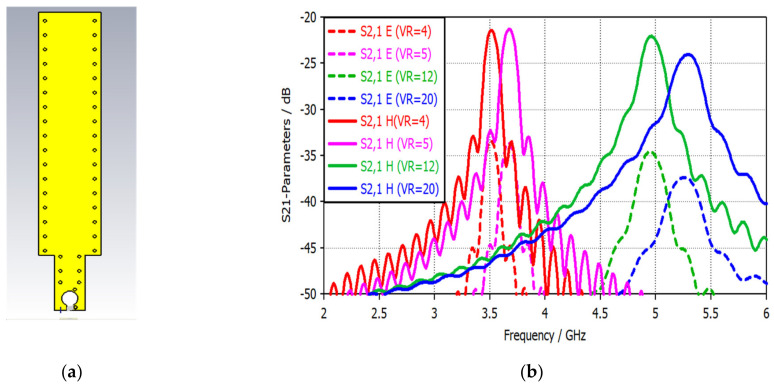
Modeling and simulation results of the via fence isolation technique: (**a**) The final geometrical shape of the magnetic probe; (**b**) isolation for the multi-narrow bands.

In this work, the first and fourth layers are made of copper and connected through vias to shield the magnetic probe against the electrical field. The vias-connected ground planes impact the isolation and field distribution and make the stripline equipotential, where any point has the same potential [34]. Moreover, the top and bottom layers have a gap in the middle of the loop at the top side, which splits the shield symmetrically and creates an open circuit for eliminating the electrical current passing on the surface due to the symmetric distribution. An unsymmetrical gap will make the probe sensitive to the tangential electrical field due to it not canceling the electrical currents flowing on the outer surface. Figure 23a presents the final geometrical shape of the switchable magnetic probe, while Figure 23b shows the isolation results for different switching states. The via and fence technique offers an isolation value of about 13 dB.

**Figure 24:**
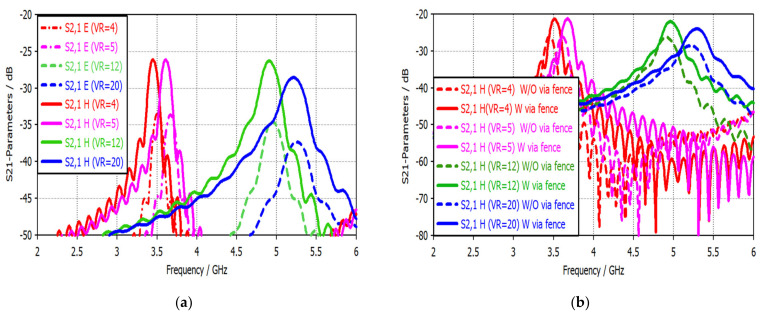
S21-parameter simulation results for different biasing voltages: (**a**) Isolation in terms of the S21-parameter without using the via fence technique; (**b**) Sensitivity enhancement resulting from using the via fence technique.

To verify the enhancement in sensitivity and isolation using the via fence technique, Figure 24a,b present the sensitivity and isolation with and without using this technique for different biasing voltage values. It is evident that the via fence technique considerably impacts isolation and sensitivity. For instance, a 5 dB isolation enhancement has been achieved using the via fence technique. On the other hand, a sensitivity enhancement of about 5 dB is obtained through critical applications. 

The sensitivity measures the level of the magnetic field distribution above the device under test. Comparing the achieved sensitivity with the previous results in [24,25,26,28] shows the main contribution of this work. The proposed probe has summed up the benefits of a large bandwidth with high sensitivity compared to the probes described in the literature reviewed, as illustrated in Table 3.

## 6. Conclusions

A high-sensitivity broadband-to-multi-narrowband switchable magnetic probe has been designed and modeled using CST EM Microwave Studio. This work deployed a new method to achieve the benefits of bandwidth and high sensitivity. Furthermore, the achieved results are necessary to level up the performance in the compliance test by utilizing two modes; a broadband mode with good sensitivity from 1 GHz to 3.4 GHz, and another mode with highly tunable sensitivity for WiMAX, Mid-band 5G, PAN, and WLAN applications. The switching process from broadband to narrowband is achieved with a PIN diode. At the same time, a reverse biasing varactor diode sweeps the operation point between these four bands. The adopted via fence technique significantly impacts the isolation and sensitivity enhancements. Moreover, the equivalent circuit of this probe has been modeled using AWR Software and shows S-parameter results that agree with the CST EM simulation results.

## Figures and Tables

**Table 1:** The overall dimensions of the structure.

NO.	1	2	3	4	5	6
PARAMETER	$L_{s}$	$L_{p}$	$L_{a}$	r	$r_{via1}$	$r_{via2}$
VALUE/MM	50	10	48.32	1.5	0.5	0.4
PARAMETER	$W_{s}$	$W_{p}$	$W_{a}$	t	S	$d_{via1}$
VALUE/MM	10	5	0.3	0.8	1.5	2

**Table 2:** Lists the diode modes with a variable DC supply.

NO.	1	2	3	4	5
VR/V	0	4	5	12	20
CAPACITOR/pF	–	0.8	0.7	0.3	0.23
PIN MODE	OFF	ON	ON	ON	ON
VARACTOR MODE	OFF	M1	M2	M3	M4
STATE/GHZ	1–6	3.5	3.75	4.8	5.2

**Table 3:** Comparison of the sensitivity enhancement achieved in the proposed work and in the literature reviewed.

Ref.	Bands/GHz	Improvement/dB
[24]	Up to 6	5
[25]	1.57	8.68
[26]	1.57	6.6
[28]	0.89, 1.7, and 2.2	7–9
This work	1–3, 3.5, 4.8 and 5.2	10

## Data Availability

Not applicable.
